# A Radiomics–Clinical Nomogram for Pre-Treatment Prediction of Neoadjuvant Chemotherapy Response in Locally Advanced Gastric Cancer

**DOI:** 10.3390/diagnostics16060945

**Published:** 2026-03-23

**Authors:** Qianzheng Zhou, Jun Xu, Qiong Li, Fengyuan Li, Hao Xu

**Affiliations:** 1 The First Clinical Medical College of Nanjing Medical University, Nanjing 211166, China; zqz20001023@icloud.com (Q.Z.); hzxuj1118@163.com (J.X.); njmu_lq@163.com (Q.L.); lfyuan@njmu.edu.cn (F.L.); 2 Department of Radiology, The First Affiliated Hospital of Nanjing Medical University, Nanjing 210029, China; 3 Department of General Surgery, The First Affiliated Hospital of Nanjing Medical University, Nanjing 210029, China

**Keywords:** radiomics, gastric cancer, neoadjuvant chemotherapy, treatment response, predictive nomogram

## Abstract

**Objective:** To develop and evaluate a nomogram integrating radiomic features from contrast-enhanced CT with clinical variables for pre-treatment predictions of the response to neoadjuvant therapy (NAT) in locally advanced gastric cancer (LAGC). **Methods:** In this retrospective multicenter study, 183 LAGC patients from the First Affiliated Hospital of Nanjing Medical University (2014–2023) were included. Radiomic features were extracted from manually delineated pre-treatment CT regions of interest. A machine learning-based predictive model combining radiomic scores and clinical data was constructed. Model performance was assessed using receiver operating characteristic (ROC) analysis, calibration curves, and decision curve analysis (DCA). **Results:** Multivariate analysis identified the radiomic score, preoperative *N* stage, and neoadjuvant regimen as independent predictors of NAT responses (all *p* < 0.05). The integrated nomogram achieved an area under the ROC curve of 0.807 and showed a moderate net benefit in DCA compared with the radiomics-only model. **Conclusions:** The radiomics–clinical nomogram demonstrates moderate predictive performance for pre-treatment stratification of NAT responses in LAGC. These findings are exploratory and hypothesis-generating, and further validation in independent cohorts is required before clinical application.

## 1. Introduction

Gastric cancer has become a significant factor threatening global health, with approximately over 1 million new cases and 650,000 deaths annually. Its geographical distribution varies significantly, with Asia, South America, and Eastern Europe being the main high-incidence regions. Notably, the number of gastric cancer cases in China accounts for nearly half of the global total [1], simultaneously increasing the disease burden on families and society. Major risk factors comprise Helicobacter pylori infection, excessive salt intake, lifestyle-related behaviors including tobacco use and alcohol drinking, as well as hereditary influences. Despite continuous advancements in diagnosis and treatment technologies, the overall prognosis for gastric cancer patients remains poor [2,3], primarily due to factors such as delayed diagnosis caused by atypical early symptoms, tumor heterogeneity, and treatment resistance.

Surgery is the cornerstone of gastric cancer treatment, particularly for locally advanced gastric cancer (LAGC). However, surgery alone has limited efficacy for LAGC, which has driven the development of multidisciplinary comprehensive treatment models. Neoadjuvant therapy (NAT) has become a standard component of LAGC treatment, as it can improve the rate of radical (R0) resection, eliminate micrometastases, and provide in vivo drug sensitivity information [4,5]. However, there are significant individual differences in patient responses to NAT: some patients experience significant tumor regression, while others exhibit treatment resistance [6,7]. For patients with poor responses, not only do they fail to benefit, but they may also endure toxic side effects and the risk of disease progression [8]. Currently, the evaluation of NAT efficacy mainly relies on postoperative pathology (such as Tumor Regression Grade, TRG scoring), which is lagging and cannot yet guide clinical decision-making before treatment.

With the rise of precision medicine, a range of innovative predictive technologies has been developed, creating new opportunities to tackle the clinical challenges described above. In the field of imaging, computed tomography (CT), as the gold standard for preoperative staging of gastric cancer, not only provides traditional morphological information but also contains deep imaging features that have become a research focus. Radiomics, by extracting high-throughput image features, can decode tumor heterogeneity and reflect its biological behavior. In the field of gastric cancer, CT-based radiomics has demonstrated potential for predicting patient prognosis [9,10] and NAT efficacy [11]. However, existing studies are mostly limited to imaging features and fail to integrate key clinical and biological information, which may affect the accuracy and stability of predictions.

Based on the aforementioned background, this study aims to develop and validate an integrated model combining CT radiomic features and clinical parameters for predicting the therapeutic responsiveness of gastric cancer patients before neoadjuvant therapy. This research holds multiple clinical values: first, it enables individualized efficacy prediction before treatment, providing a basis for clinical decision-making; second, it helps identify patients with treatment resistance, allowing for the formulation of alternative treatment plans, thereby avoiding ineffective treatment and its associated toxic side effects and economic burdens.

## 2. Materials and Methods

**Figure 1:**
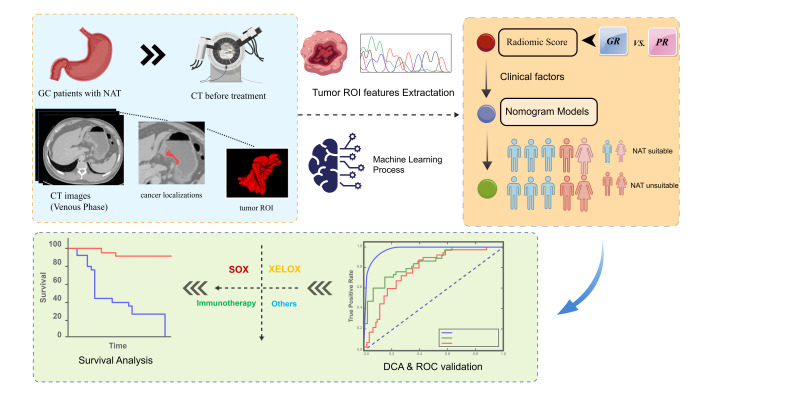
Schematic overview of the analytical pipeline used in this study.

The subjects of this study comprised 183 gastric cancer patients who underwent neoadjuvant therapy at the First Affiliated Hospital of Nanjing Medical University between January 2014 and September 2023. The inclusion criteria were as follows: (a) gastric cancer confirmed by histopathological examination; (b) gastrectomy and lymph node dissection performed after neoadjuvant therapy, with post-operative pathological confirmation of the neoadjuvant therapy regression grade; (c) having undergone contrast-enhanced CT scan before treatment. Exclusion criteria included: (a) inability to identify the primary tumor on CT or poor CT image quality; (b) concurrent presence of other malignant tumors; (c) absence of pre-treatment CT images; and (d) missing other clinical data. Based on an expected AUC value of 0.8 for the predictive model, with α = 0.05 and β = 0.2, the sample size was preliminarily estimated using the ROC curve sample size calculation formula to be approximately 130 cases. The present study included 183 patients, meeting the minimum sample size requirement. This study was approved by the hospital’s ethics review committee (Approval No.: 2024-SR-180), and all patients provided informed consent. The schematic overview of the analytical pipeline used in this study is illustrated in Figure 1. This study followed the Transparent Reporting of a multivariable prediction model for Individual Prognosis or Diagnosis (TRIPOD) statement for prediction model development and validation. Additionally, radiomics analysis was conducted in accordance with the Image Biomarker Standardization Initiative (IBSI) guidelines to ensure reproducibility and standardization of feature calculation.

The clinical baseline characteristics of the patients included age, gender, body mass index (BMI), tumor differentiation, carcinoembryonic antigen (CEA), alpha-fetoprotein (AFP), and carbohydrate antigen 19-9 (CA 19-9). Tumor infiltration depth, lymph node metastasis stage, and pathological stage were all extracted from clinical records according to the 8th edition of the American Joint Committee on Cancer (AJCC) TNM staging system.

All enrolled patients received 2 to 6 cycles of neoadjuvant chemotherapy, including standard SOX regimen, XELOX regimen, combination immunotherapy (Immu), and other regimens adjusted according to the patient’s condition. Before surgery, treatment dosages or cycles were adjusted for each patient according to therapeutic response and individual tolerance. The decision regarding surgical timing was guided by the effectiveness of neoadjuvant therapy, as assessed through symptom improvement, normalization or persistent decline in tumor marker levels, and radiologic evidence of tumor and suspected metastatic lymph node shrinkage on CT or MRI. All patients completed a minimum of two cycles of neoadjuvant treatment, with no early discontinuation or modification of therapeutic agents. The interval between the final treatment session and surgical intervention did not exceed one month.

The evaluation of neoadjuvant treatment efficacy was based on postoperative pathological reports. According to the Mandard tumor regression grade (TRG) system used in our center’s pathological reports, specimens after neoadjuvant therapy were classified into grades 0 to 5. A post-neoadjuvant therapy grade of 0 indicates pathological complete response (pCR), with no viable tumor cells identified; a grade of 5 indicates complete ineffectiveness, with no change or progression of the tumor. For this study, patients with neoadjuvant therapy grade 0–1 were categorized into the Good Response (GR, *n* = 55) group, and those with grade 2–5 were categorized into the Poor Response (PR, *n* = 128) group. This dichotomization was chosen based on clinical experience and established prognostic evidence, as patients with grade 0–1 consistently demonstrate significantly better survival outcomes compared to those with grade 2–5. Grade 0 approximately correspond to Becker regression grade 1a, grade 1 corresponds to Becker regression grade 1b, grade 2 and 3 corresponds to Becker regression 2, and grades 4 and 5 approximately correspond to Becker regression grade 3.

All patients underwent preoperative contrast-enhanced CT at our hospital using a 128-slice scanner (SOMATOM Definition AS+, Siemens, Forchheim, Germany). Patients fasted for 4–6 h before the examination. Scans were performed in the supine position from the diaphragmatic dome to the pelvic floor during breath-hold. Scanning parameters were: tube voltage 120 kVp, tube current 180 mAs, helical pitch 0.5 s, matrix 512 × 512, and reconstruction slice thickness 1.5 mm. Non-contrast imaging was followed by contrast-enhanced scanning, with a non-ionic contrast agent injected via an antecubital vein using a power injector at 1.5 mL/kg and 3.5 mL/s. Arterial and venous phase scans were initiated 20 s and 50 s after bolus triggering (trigger threshold: 100 HU in the abdominal aorta). Tumor ROIs were independently delineated by two radiologists blinded to treatment outcomes; inter-observer reproducibility was assessed in a random subset of 30 patients, retaining features with ICC > 0.86. The scanner and acquisition protocol remained unchanged throughout the study. Radiomics features were extracted using PyRadiomics (version 3.1.0), with all images resampled to 1 × 1 × 1 mm^3^ via B-spline interpolation and gray-level discretization using a fixed bin width of 25 HU. Data preprocessing included upsampling to balance classes and normalization to zero mean and unit variance. Feature selection was performed using LASSO regression with ten-fold cross-validation to identify the most relevant features for modeling.

This study employed four machine learning methods for model development: Support Vector Machine (SVM), Random Forest (RF, with the number of trees increased to 500), Logistic Regression (LR), and Decision Tree (Tree). The machine learning process was conducted in R (version 4.4.2), utilizing the “caret”, “randomForest”, “e1071”, and “rpart” R packages for building the respective models. The dataset was split into training and validation sets in a 7:3 ratio.

Receiver Operating Characteristic (ROC) curve analysis was performed. The discriminatory performance of the different models was quantified using the Area Under the Curve (AUC), sensitivity, and specificity. The Delong test was used to compare the differences between the AUCs of two models.

Statistical analyses were conducted using SPSS software (version 26.0) and R (version 4.4.2). Continuous clinical variables were compared between groups using the Mann–Whitney U test, whereas categorical variables were analyzed with the Chi-square test or Fisher’s exact test, as appropriate. ROC and decision curve analyses were performed in R. Calibration curves were generated with the rms package (version 6.2), and decision curves were constructed using the rmda package (version 1.6). All statistical tests were two-tailed, and a *p* value of less than 0.05 was considered statistically significant.

## 3. Results

### 3.1. Analysis of Baseline Characteristics

Table 1 presents the baseline characteristics of the 183 gastric cancer patients who received neoadjuvant therapy. Among them, the proportion of patients in the Good Response (GR) group was 30.1%, and the proportion in the Poor Response (PR) group was 69.9%. Among all baseline indicators, only the clinical T (cT) stage showed a statistically significant difference between the GR and PR groups (*p* = 0.014), while the remaining indicators showed no significant differences. The dataset was divided into training and validation sets in a 7:3 ratio. The division results are shown in Table 2, with 128 cases in the training set and 55 cases in the validation set. In the training set, the GR proportion was 28.9% and the PR proportion was 71.1%; in the validation set, the GR proportion was 32.7% and the PR proportion was 67.3%. No significant difference was observed in the GR/PR ratio between the training and validation cohorts, and none of the remaining variables showed statistically significant differences (in Figure 1). The median follow-up time for the entire cohort was 57 months. By the end of follow-up, 39 patients (21.3%) had died, and 14 patients (7.7%) had experienced disease recurrence.

### 3.2. Survival Analysis of the Patient Cohort

**Figure 2:**
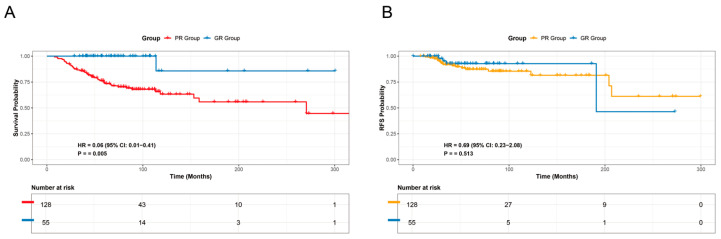
Survival curves in the neoadjuvant therapy cohort: (**A**) Overall survival (OS) difference between the responders (GR, *n* = 55) and non-responders (PR, *n* = 128). (**B**) Recurrence-free survival (RFS) difference between the GR (*n* = 55) and PR (*n* = 128) groups.

The Kaplan–Meier method was employed, followed by the log-rank test, to compare the overall survival (OS) and recurrence-free survival (RFS) between the GR (Good Response) and PR (Poor Response) groups within the neoadjuvant therapy patient cohort. The results indicated a statistically significant difference in OS between the GR and PR groups. However, no statistically significant difference in RFS was observed between the two groups (Figure 2).

### 3.3. Radiomics Feature Selection and Model Construction

**Figure 3:**
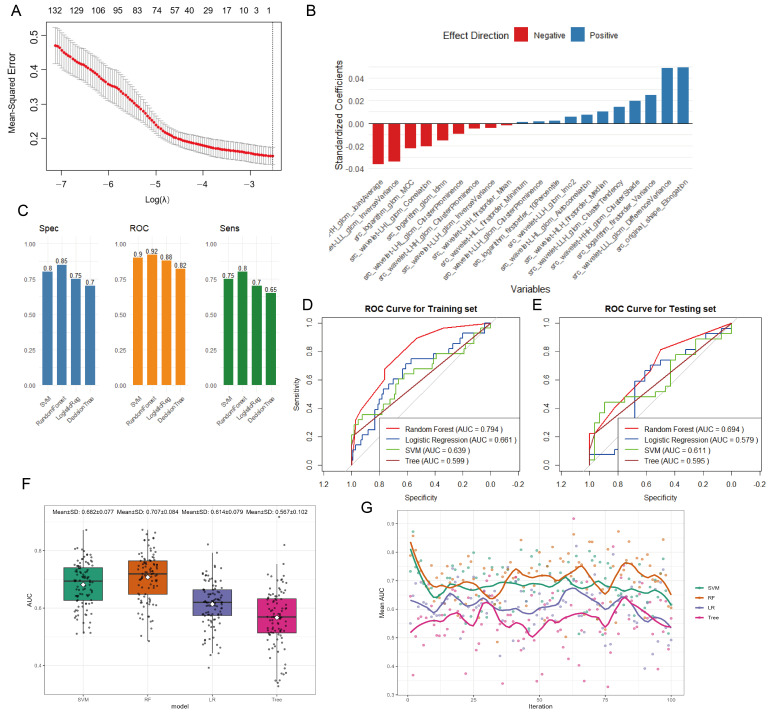
Development and performance validation of the machine learning models. (**A**) Performance of the LASSO regression model across different regularization parameters (λ). (**B**) Top 20 most relevant features selected. (**C**) Feature importance ranking from the random forest model. (**D**) Performance comparison of the four models in Sensitivity (Sens), Specificity (Spec), and AUC. (**E**,**F**) ROC curves of the four models in the training (**E**) and validation (**F**) sets, respectively. (**G**) Bootstrap validation (100 replicates) of model performance. Box plot of optimism-corrected AUC (mean ± SD).

Following feature screening via LASSO regression, an appropriate λ value (approximately 0.080) was selected to identify the top 20 most relevant features for machine learning model development (Figure 3A,B). Figure 3C, using the RF model as an example, displays these top 20 features. (Features in Figure 3B are listed in descending order of importance: src_original_shape_Elongation,src_wavelet-LLL_glcm_DifferenceVariance,src_wavelet-LHH_glcm_JointAverage,src_wavelet-LLL_glcm_InverseVariance,src_logarithm_firstorder_Variance,src_logarithm_glcm_MCC,src_wavelet-LHL_glcm_Correlatio,src_wavelet-HHH_glcm_ClusterShade,src_logarithm_glcm_Idmn,src_wavelet-LLH_glcm_ClusterTendency,src_wavelet-HLH_firstorder_Median,src_wavelet-LHL_glcm_ClusterProminence,src_wavelet-LHL_glcm_Autocorrelation,src_wavelet-LLH_glcm_Imc2,src_wavelet-LHH_glcm_ClusterProminence,src_wavelet-LLH_glcm_InverseVariance,src_logarithm_firstorder_10Percentile,src_wavelet-LHH_firstorder_Mean,src_wavelet-LLH_glcm_ClusterProminence,src_wavelet-HLL_firstorder_Minimum).

### 3.4. Predictive Performance of Machine Learning Models

After model construction, the performance of the four models was compared based on three metrics: the Area Under the ROC Curve (AUC), specificity, and sensitivity (Figure 3D). The Random Forest (RF) model demonstrated superior performance in all three aspects—AUC, specificity, and sensitivity—compared to the other models. Subsequently, ROC curves were plotted for both the training set and the validation set (Figure 3E,F) for comparison. The RF model showed better predictive performance than the other models in both the training and validation sets.

To mitigate potential bias arising from the small sample size and ensure rigorous validation, we employed Bootstrap resampling with 100 repetitions. To prevent data leakage, the entire modeling process, including radiomics feature selection and model tuning, was repeated within each bootstrap iteration. The model’s performance was then assessed on the out-of-bag samples (Figure 3G,H). Ultimately, the RF model’s average AUC value and optimism-corrected AUC value were superior to those of the other models. The average optimism-corrected AUC and AUC values for RF were 0.707 and 0.920, respectively; for SVM, they were 0.682 and 0.900; for LR, they were 0.614 and 0.880; and for Tree, they were 0.567 and 0.820.

The DeLong test was applied to evaluate performance differences among the models, and the corresponding results are shown in Table 3. Significant differences were identified between the RF model and each of the remaining models (RF vs. SVM, *p* = 0.004; RF vs. Tree, *p* = 0.001; RF vs. LR, *p* = 0.008). However, performance comparisons among SVM, Decision Tree, and Logistic Regression revealed no statistically significant differences.

### 3.5. Multivariate Analysis Incorporating Clinical Data

**Figure 4:**
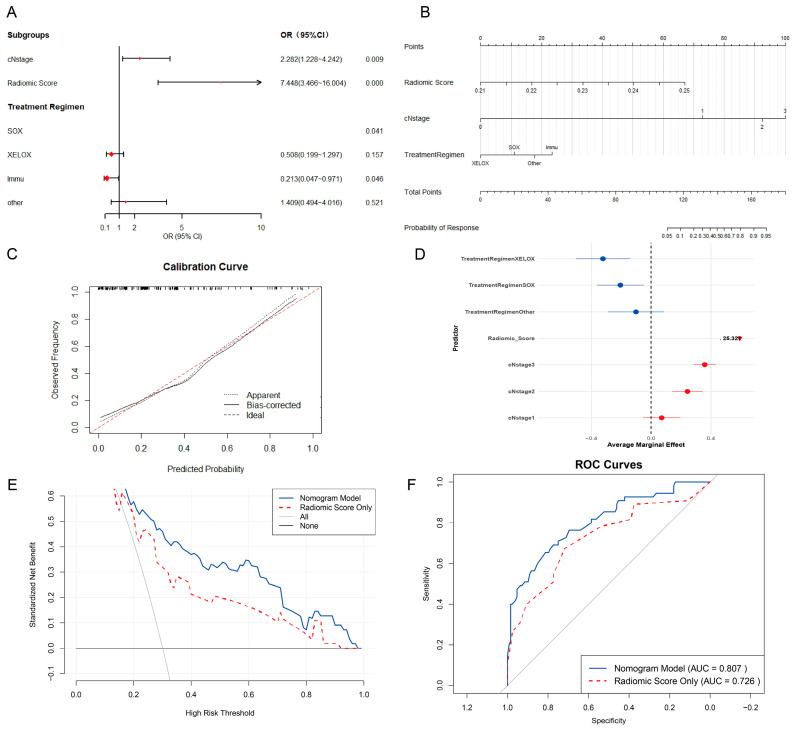
Development and performance validation of the nomogram model (**A**) Forest plot displaying the results of the multivariable analysis. (**B**) Nomogram for predicting response to neoadjuvant therapy. (**C**) Calibration curve of the nomogram. (**D**) Marginal contribution of each variable included in the nomogram to individual predictions. (**E**,**F**) DCA (**E**) and ROC (**F**) curves of the nomogram model and the radiomic score only model in the entire cohort, respectively. The binary logistic regression models in (**A**,**F**) were assessed by the Hosmer–Lemeshow test (*p* = 0.993), indicating good agreement between predictions and observations.

Based on the analysis of the model prediction results, the RF model was selected as the basis for calculating the radiomics score (Table 3). A Logistic regression model was then constructed by combining this radiomics score with other baseline characteristics. Variables showing a statistically significant association with neoadjuvant treatment efficacy in the univariate analysis—specifically, neoadjuvant immunotherapy regimen (*p* = 0.046), radiomics score (*p* < 0.001), and clinical N (cN) stage (*p* = 0.009)—were incorporated into the multivariate regression analysis (Table 4). The results revealed that the neoadjuvant treatment regimen, radiomics score, and cN stage were all independent risk factors (Figure 4A).

### 3.6. Development of a Nomogram Model and Performance Validation and Comparison

Based on the results of the multivariate analysis, a nomogram prediction model was constructed (Figure 4B), and its corresponding calibration curve was plotted (Figure 4C). This nomogram model demonstrated good discriminative performance, achieving an Area Under the ROC Curve (AUC) of 0.807 (Figure 4F). The Decision Curve Analysis (DCA) results indicated that the clinical net benefit of the nomogram model was significantly superior to that of the radiomics-only model across various probability thresholds (Figure 4E). Additionally, a Delong test was performed, yielding a *p*-value of 0.0203, suggesting a statistically significant difference in the predictive performance between the two models.

Considering that immunotherapy was identified as an independent risk factor, we performed a stratified analysis by treatment regimen to exclude the potential confounding effect of immunotherapy. ROC curves were plotted for the immunotherapy group and the chemotherapy group (SOX/XELOX) separately (Figure S1). The model achieved an AUC of 0.776 in the chemotherapy group and 0.816 in the immunotherapy group. The DeLong test showed no significant difference between the two groups (*p* = 0.602), indicating comparable predictive performance across different treatment regimens.

Cutoff values for the nomogram and radiomics models were determined by the Youden index, yielding thresholds of 0.355 and 0.257, respectively. Patients were subsequently stratified according to these scores. Survival outcomes, including OS and RFS, were compared across groups using the Kaplan–Meier method in conjunction with the log-rank test.

**Figure 5:**
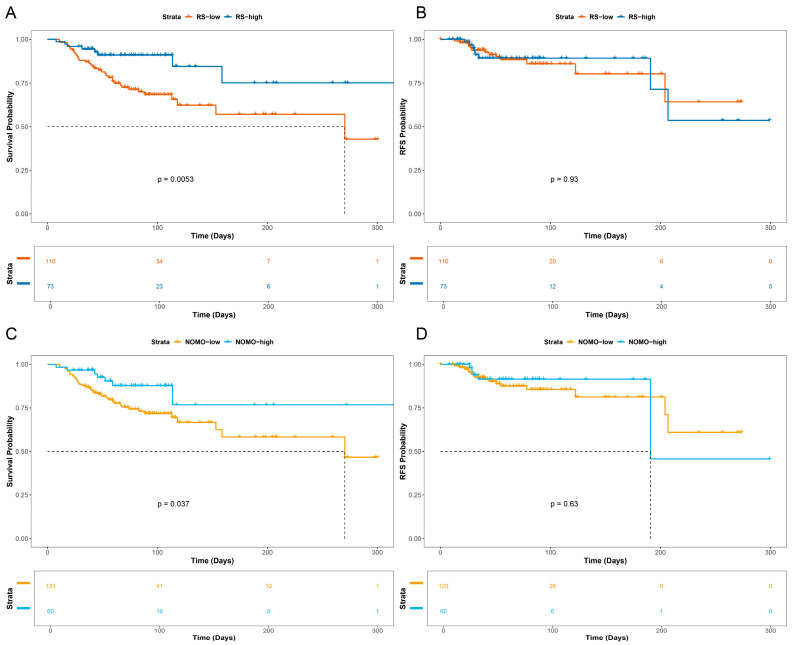
Predictive performance of the nomogram and radiomics models for patient survival (**A**) Difference in OS between high and low radiomic score groups. (**B**) Difference in RFS between high and low radiomic score groups. (**C**) Difference in OS between groups stratified by the nomogram model. (**D**) Difference in RFS between groups stratified by the nomogram model.

The survival analysis revealed that both the nomogram model and the radiomics model effectively predicted OS (*p* < 0.05; Figure 5A,C). However, neither model achieved statistical significance in predicting RFS (*p* > 0.05; Figure 5B,D).

### 3.7. SHAP Analysis of Radiomics Features

**Figure 6:**
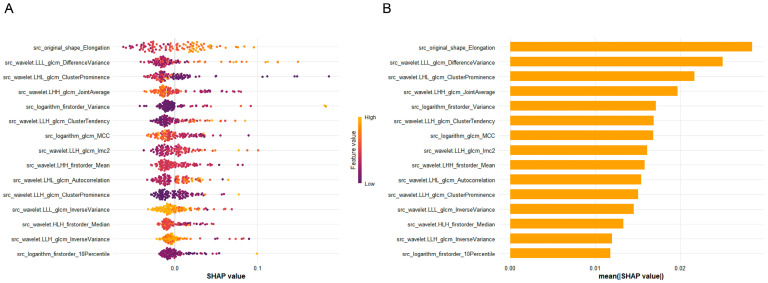
SHAP analysis of radiomic features (**A**) Distribution of feature importance based on SHAP values. The x-axis indicates the direction and magnitude of each feature’s contribution to the model predictions, while the y-axis lists the different features. (**B**) Ranking of features by their mean absolute SHAP values.

To better illustrate how specific radiomic features influenced the model’s predictions, we conducted a Shapley Additive Explanations (SHAP) analysis based on the features used in model construction. As shown in Figure 6, the SHAP summary plot and bar plot (Figure 6A,B, respectively) revealed that both morphological and textural features contributed substantially to the model output. Among them, Elongation exhibited the highest overall importance, followed by several wavelet-based textural features derived from the Gray Level Co-occurrence Matrix (GLCM). The distribution of SHAP values further indicated that higher values of these key features were generally associated with a positive contribution to the model’s predictions. This alignment between feature importance rankings and their directional impact enhances the interpretability of the model and clarifies the link between visual patterns in the SHAP figures and the corresponding textual descriptions. (Top5 feature: src_original_shape_Elongation, src_wavelet-LLL_glcm_DifferenceVariance, src_wavelet-LLL_glcm_ClusterProminence, src_wavelet-LHH_glcm_JointAverage,src_logarithm_firstorder_Variance).

## 4. Discussion

This study developed a machine learning-based nomogram model incorporating radiomics features and clinical parameters to predict the efficacy response of gastric cancer patients to neoadjuvant therapy (NAT). Compared to traditional radiomics-only models, this integrated model, by fusing radiomics features with key clinical variables, demonstrated improved predictive performance. While the model demonstrated promising internal performance, its clinical applicability requires further confirmation through external validation. It may assist in clinical decision-making through an intuitive nomogram format, thereby offering potential to inform individualized treatment strategies and maximize patient benefit from neoadjuvant therapy, although prospective validation in independent cohorts is necessary to establish its generalizability and clinical utility.

Previous studies have demonstrated that neoadjuvant therapy (NAT) provides clear survival benefits for patients with locally advanced gastric cancer (LAGC), significantly increasing the R0 resection rate, reducing the risk of postoperative recurrence, and improving long-term prognosis [12,13,14]. However, clinical practice shows that a considerable proportion of patients exhibit poor response to NAT, and some may even experience disease progression, leading to treatment delays and unnecessary toxic burden [15]. Early identification of such patients is crucial, yet reliable predictive tools are still lacking. Therefore, accurately identifying potential non-responders before treatment and developing a reliable non-invasive predictive tool for this purpose are essential for achieving treatment individualization, avoiding the waste of medical resources, and improving patient quality of life.

Radiomics has been widely investigated in oncology for high-throughput quantitative feature extraction, with prior studies demonstrating its utility in treatment stratification [16], metastasis prediction [17], and NAT response assessment [18,19]. Machine learning–based radiomics models have shown promising discrimination in gastric cancer. For example, Zhang et al. [20] reported AUCs of 0.848, 0.802, and 0.751 in training, internal validation, and external validation cohorts, respectively, illustrating the expected attenuation of performance in independent datasets. In the present study, the apparent AUC of the nomogram was 0.807. However, bootstrap-based optimism correction reduced the model AUC to 0.707, providing a more conservative estimate of discrimination. This difference suggests model optimism, which is common in high-dimensional radiomics analyses. An AUC around 0.70 indicates moderate discrimination for complex biological endpoints such as pathological response. Given the absence of external validation, the findings should be interpreted cautiously. The model is best regarded as exploratory and hypothesis-generating, pending confirmation in independent multicenter cohorts.

Multivariate logistic regression analysis revealed that preoperative clinical N (cN) stage, whether receiving neoadjuvant immunotherapy, and the radiomics score were independent risk factors influencing patient response to NAT. In the marginal contribution analysis of variables, the predictive weight of the radiomics score was significantly higher than that of other variables. The predictive capability of radiomics for NAT efficacy is supported by existing research [19,20].

Regarding the impact of cN stage on treatment efficacy, Wen et al. [21] indicated that both the cN and clinical T (cT) stage are independent factors influencing NAT efficacy in patients with advanced rectal cancer. In this study, baseline analysis showed that although cT stage differed significantly between the GR (Good Response) and PR (Poor Response) groups (*p* = 0.0138), only the cN stage retained statistical significance in the multivariate analysis (*p* = 0.009). This discrepancy may stem from differences in tumor biological characteristics or heterogeneity within the study population.

Previous studies have reported that neoadjuvant immunotherapy may improve long-term outcomes in gastric cancer compared with conventional chemotherapy [22]. In our cohort, immunotherapy was identified as an independent predictor of pathological response. However, treatment allocation was determined by clinical judgment, which may have been influenced by factors such as tumor burden, patient performance status, or other clinical characteristics, rather than by randomization; consequently, residual confounding cannot be excluded. To further explore treatment effects, regimen-stratified ROC analyses were performed (Figure S1), showing AUCs of 0.776 for chemotherapy and 0.816 for immunotherapy, with no significant difference between groups (DeLong test, *p* = 0.602). While these results indicate relatively stable model performance across regimens, it is important to note that this stability does not eliminate confounding by indication, as treatment selection bias is inherent to the retrospective design. These findings suggest that the predictive contribution of radiomics features mainly reflects intrinsic tumor imaging phenotypes rather than treatment-specific effects, though prospective studies with more balanced treatment allocation are needed to clarify interactions between tumor phenotype and therapy.

SHAP analysis identified several radiomics features—such as src_original_shape_Elongation, src_wavelet-LLL_glcm_DifferenceVariance, and src_wavelet-LHH_glcm_JointAverage—as major contributors to model output. These features primarily capture tumor morphology and textural heterogeneity, which may reflect variations in tumor architecture and spatial complexity. While such imaging characteristics could be associated with underlying biological heterogeneity, these interpretations remain speculative, as no molecular or mechanistic validation was performed in this research. Prior studies have suggested links between immunoregulatory markers such as SIGLEC-15 and PD-L1 expression and aggressive tumor behavior [23]; however, direct biological validation linking specific radiomics features to molecular pathways was beyond the scope of the present study. Therefore, the biological implications of the identified features should be considered hypothesis-generating, and future investigations integrating radiomics with genomic or immunohistochemical profiling are needed to further elucidate potential mechanistic associations.

Currently, the gold standard for evaluating NAT efficacy remains postoperative pathological examination. While highly accurate, it suffers from significant time lag, making it difficult to provide a basis for real-time adjustments during treatment, thereby limiting its clinical value. Research by Deng et al. [24] demonstrated that radiomics can be used for post-treatment efficacy assessment and surgical timing selection. Their method, based on the dynamic changes in imaging features during treatment, can effectively monitor treatment response and guide surgical decision-making.

In contrast, the predictive model proposed in this study focuses on the pre-treatment phase, offering the potential for earlier identification of patients who may benefit from NAT. In doing so, it could help avoid unnecessary toxicity and economic burden associated with ineffective therapy and may reduce the risk of tumor progression due to treatment delays. These potential advantages are consistent with the goals of precision medicine, though they require prospective validation.

Previous studies, such as that by Zhong et al. [25], have leveraged deep learning to construct nomograms that effectively predict the response of metastatic lymph nodes to neoadjuvant therapy (NAT) in locally advanced gastric cancer. Building on this approach, our study established a radiomics score derived from machine learning algorithms and integrated it with clinical parameters to develop a clinical-radiomics combined nomogram model. This integrated model offers two potential advantages: first, by incorporating variables such as clinical N (cN) stage and immunotherapy status, it demonstrates improved predictive performance compared to single-modality models in this cohort; second, its presentation as a visual nomogram may enhance clinical interpretability. If validated in independent cohorts, such a tool could potentially contribute to risk stratification frameworks. At present, however, it should be regarded as a preliminary decision-support concept rather than a clinically deployable instrument.

Although the primary objective of this study was to predict pathological response (TRG), we further explored the association between model-derived risk stratification and survival outcomes. In this exploratory analysis, both the nomogram and radiomics-only models suggested a potential trend in stratifying Overall Survival (OS), although this finding requires further validation. However, these analyses should be considered exploratory, as the models were not originally developed as prognostic tools. Neither model showed a significant association with Recurrence-Free Survival (RFS). This discrepancy underscores the distinction between predictive modeling of treatment response and long-term prognostic assessment. Pathological response may not directly translate into durable recurrence control, particularly in the context of heterogeneous post-treatment management. In this exploratory cohort, Decision Curve Analysis suggested that the nomogram model may provide a higher standardized net benefit across a range of risk thresholds compared with the radiomics-only model. Nevertheless, these findings should be interpreted cautiously, given the limited sample size and absence of external validation. The potential clinical utility of survival stratification requires confirmation in independent cohorts specifically designed for prognostic evaluation.

This study has several limitations. First, its retrospective single-center design with a limited sample size and extended enrollment period is prone to selection bias, despite bootstrap resampling for internal validation. The absence of an independent external validation cohort means generalizability remains unconfirmed; temporal or multi-center validation is needed. Second, pathological assessment by multiple physicians may have introduced variability; central review using standardized criteria (e.g., Becker) is warranted. Third, the retrospective design precluded strict control of treatment regimens, with reliance on medical records introducing potential inaccuracy. Fourth, excluding patients who did not undergo surgery (due to progression or toxicity) limits applicability to advanced disease. Fifth, only routine blood tests and traditional tumor markers were analyzed; molecular features (e.g., HER2, PD-L1) that may influence response were unavailable and should be included prospectively. Sixth, the lack of significant RFS prediction, unlike OS, suggests confounding by post-treatment interventions, limiting clinical interpretation for recurrence. Seventh, the apparent performance (AUC = 0.807) likely overestimates true discrimination, as the optimism-corrected AUC was substantially lower (0.707), underscoring the need for cautious interpretation and external validation. The substantial gap between apparent and optimism-corrected AUC underscores the risk of overfitting inherent to high-dimensional radiomics modeling.

On a methodological level, heterogeneity in CT scanning parameters might affect the stability of radiomics features. Although tumor ROIs were independently delineated by two radiologists with a high intraclass correlation coefficient (ICC = 0.86), inter-observer variability remains difficult to completely eliminate, especially for lesions with poorly defined borders or suboptimal image quality.

## 5. Conclusions

This study developed a machine learning-based nomogram integrating radiomics and clinical features to predict neoadjuvant therapy response in gastric cancer. The model showed moderate predictive performance (AUC = 0.807) and stable discrimination across treatment subgroups. Survival analyses were exploratory, with limited predictive ability for recurrence-free survival.

The novelty lies in combining radiomic and clinical data for pre-treatment prediction, providing a methodological foundation for future research. As a single-center retrospective study without external validation, the findings require confirmation in multicenter prospective cohorts. The nomogram represents an exploratory approach for pre-treatment stratification, pending further validation.

## Figures and Tables

**Table 1:** Comparison of basic information between the two groups of patients.

	GR Group	PR Group	*p*
Variable			
	(*n* = 55)	(*n* = 128)	
Sex [n (%)]			
Female	12 (21.8)	29 (22.7)	0.000
Male	43 (78.2)	99 (77.3)	
Age [n (%)]			
>60 years	25 (45.5)	75 (58.6)	0.140
≤60 years	30 (54.5)	53 (41.4)	
BMI [kg/m^2^]			
Mean ± sd	23.3 ± 2.90	23.0 ± 2.69	0.437
M (P_25_, P_75_)	22.9 (17.6, 31.4)	22.7 (17.6, 33.1)	
Treatment regimen [n (%)]			
Immunotherapy	17 (30.9)	21 (16.4)	0.082
other	13 (23.6)	28 (21.9)	
SOX	22 (40.0)	62 (48.4)	
XELOX	3 (5.5)	17 (13.3)	
cTstage [n (%)]			
1	0	2 (1.6)	0.014
2	0	4 (3.1)	
3	15 (27.3)	36 (28.1)	
4a	33 (60.0)	84 (65.6)	
4b	7 (12.7)	2 (1.6)	
cNstage [n (%)]			
0	0	3 (2.3)	0.056
1	1 (1.8)	15 (11.7)	
2	14 (25.5)	38 (29.7)	
3	40 (72.7)	72 (56.3)	
WBC [n×10^9^/L]			
Mean ± sd	6.54 ± 2.26	6.22 ± 1.89	0.354
M (P_25_, P_75_)	5.89 (2.56, 13.8)	5.83 (2.57, 14.0)	
RBC [n×10^12^/L]			
Mean ± sd	4.07± 0.801	4.28 ± 0.589	0.078
M (P_25_, P_75_)	4.03 (2.29, 5.73)	4.32 (2.77, 6.01)	
PLT [n×10^9^/L]			
Mean ± sd	239 ± 93.2	223 ± 70.8	0.262
M (P_25_, P_75_)	223 (107, 500)	220 (61.0, 440)	
LYM [n×10^9^/L]			
Mean ± sd	1.62 ± 0.565	1.63 ± 0.646	0.863
M (P_25_, P_75_)	1.54 (0.320, 3.49)	1.54 (0.570, 4.70)	
NEU [n×10^9^/L]			
Mean ± sd	4.21 ± 1.96	4.55 ± 6.05	0.559
M (P_25_, P_75_)	3.72 (1.35, 12.1)	3.67 (1.56, 69.6)	
HB [g/L]			
Mean ± sd	118 ± 26.8	124 ± 22.6	0.114
M (P_25_, P_75_)	120 (58.0, 170)	129 (51.0, 175)	
Ca [mmol/L]			
Mean ± sd	2.23 ± 0.110	2.23 ± 0.152	0.804
M (P_25_, P_75_)	2.21 (2.02, 2.47)	2.24(1.07, 2.50)	
Mg [mmol/L]			
Mean ± sd	0.863 ± 0.0652)	0.890 ± 0.141	0.071
M (P_25_, P_75_)	0.860 (0.670, 0.990)	0.880 (0.690, 2.28)	
CEA [n (%)]			
>5 ng/mL	17 (30.9)	36 (28.1)	0.839
≤5 ng/mL	38 (69.1)	92 (71.9)	
AFP [n (%)]			
>7 ng/mL	9 (16.4)	15 (11.7)	0.539
≤7 ng/mL	46 (83.6)	113 (88.3)	
CA199 [n (%)]			
>30 U/mL	17 (30.9)	27 (21.1)	0.216
≤30 U/mL	38 (69.1)	101 (78.9)	

**Table 2:** Comparison of grouped data between the training set and the validation set.

Variable	Training Set	Validation Set	*p*
	(*n* = 128)	(*n* = 55)	
Sex [n (%)]			
Female	33 (25.8)	8 (14.5)	0.139
Male	95 (74.2)	47 (85.5)	
AGE [n (%)]			
>60 years	69 (53.9)	31 (56.4)	0.885
≤60 years	59 (46.1)	24 (43.6)	
BMI [kg/m^2^]			
Mean ± sd	23.0 ± 2.91	23.2 ± 2.33	0.761
M (P_25_, P_75_)	22.7 (17.6, 33.1)	22.8 (17.6, 30.7)	
Missing	10 (7.8)	7 (12.7)	
Treatment Regimen [n (%)]			
Immunotherapy	31 (24.2)	7 (12.7)	0.315
other	26 (20.3)	15 (27.3)	
SOX	58 (45.3)	26 (47.3)	
XELOX	13 (10.2)	7 (12.7)	
cTstage [n (%)]			
1	2 (1.6)	0	0.364
2	4 (3.1)	0	
3	33 (25.8)	18 (32.7)	
4a	84 (65.6)	33 (60.0)	
4b	5 (3.9)	4 (7.3)	
cNstage [n (%)]			
0	3 (2.3)	0	0.249
1	14 (10.9)	2 (3.6)	
2	36 (28.1)	16 (29.1)	
3	75 (58.6)	37 (67.3)	
WBC [n×10^9^/L]			
Mean ± sd	6.17 ± 1.88	6.63 ± 2.26	0.190
M (P_25_, P_75_)	5.71 (2.56, 14.0)	6.16 (2.57, 13.8)	
RBC [n×10^12^/L]			
Mean ± sd	4.19 ± 0.665)	4.29 ± 0.665	0.320
M (P_25_, P_75_)	4.19(2.48, 6.01)	4.37 (2.29, 5.73)	
PLT [n×10^9^/L]			
Mean ± sd	228 ± 76.2	226 ± 83.5	0.879
M (P_25_, P_75_)	221 (105, 473)	220 (61.0, 500)	
LYM [n×10^9^/L]			
Mean ± sd	1.61 (0.635)	1.66 (0.593)	0.585
M (P_25_, P_75_)	1.53 (0.320, 4.70)	1.59(0.670, 4.10)	
NEU [n×10^9^/L]			
Mean ± sd	4.51 ± 6.05	4.31 ± 1.98	0.743
M (P_25_, P_75_)	3.65 (1.35, 69.6)	3.74 (1.56, 12.1)	
HB [g/L]			
Mean ± sd	122 ± 25.2	124 ± 21.5	0.477
M (P_25_, P_75_)	124 (51.0, 175)	128(67.0, 170)	
Ca [mmol/L]			
Mean ± sd	2.23 ± 0.152	2.23 ± 0.111	0.759
M (P_25_, P_75_)	2.24 (1.07, 2.50)	2.22 (2.03, 2.48)	
Mg [mmol/L]			
Mean ± sd	0.887 ± 0.141	0.871 ± 0.0683	0.295
M (P_25_, P_75_)	0.870 (0.670, 2.28)	0.870(0.720, 0.990)	
CEA [n (%)]			
>5 ng/mL	35 (27.3)	18 (32.7)	0.577
≤5 ng/mL	93 (72.7)	37 (67.3)	
AFP [n (%)]			
>7 ng/mL	16 (12.5)	8 (14.5)	0.891
≤7 ng/mL	112 (87.5)	47 (85.5)	
CA199 [n(%)]			
>30 U/mL	32 (25.0)	12 (21.8)	0.785
≤30 U/mL	96 (75.0)	43 (78.2)	
Response [n (%)]			
GR	37 (28.9)	18 (32.7)	0.733
PR	91 (71.1)	37 (67.3)	

**Table 3:** Results of the DeLong test among models.

Model	Random Forest	SVM	Tree	Logistic Regression
Random forest	NA	0.004	0.001	0.008
SVM	0.004	NA	0.515	0.328
Decision tree	0.001	0.515	NA	0.467
Logistic regression	0.008	0.328	0.467	NA

The numbers in the table are the p-values for comparisons between the two models. NA indicates that comparison between identical models is not applicable.

**Table 4:** Univariable and multivariable analysis of response to neoadjuvant therapy.

Variable	Univariate Analysis				Multivariate Analysis		
		OR	95.0% CI	*p*	OR	95.0% CI	*p*
Sex	Male	1.050	0.490~2.249	0.901			
	Female						
Age	>60 years	0.589	0.312~1.113	0.103			
	≤60 years						
Treatment regimen	SOX			0.940			0.041
	XELOX	0.497	0.133~1.862	0.300	0.508	0.199~1.297	0.157
	Immu	2.281	1.022~5.095	0.044	0.213	0.047~0.971	0.046
	other	1.308	0.577~2.965	0.520	1.409	0.494~4.016	0.521
BMI		1.051	0.931~1.186	0.420			
CEA		0.997	0.990~1.005	0.461			
AFP		1.000	0.998~1.002	0.861			
CA199		1.000	0.998~1.002	0.674			
Radiomic score		5.253	2.655~10.394	0.000	7.448	3.466~16.004	0.000
cT stage	1	1.466	0.803~2.676	0.213			
	2						
	3						
	4						
cN stage	0	2.053	1.189~3.543	0.010	2.282	1.228~4.242	0.009
	1						
	2						
	3						

## Data Availability

The original contributions presented in this study are included in the Supplementary Material. Further inquiries can be directed to the corresponding author.

## Supplementary Materials

The following supporting information can be downloaded at: https://www.mdpi.com/article/10.3390/diagnostics16060945/s1, Figure S1. ROC curves of the nomogram model stratified by treatment group. Data S1. stable1.
