# Supplementary figures and images for: A Radiomics–Clinical Nomogram for Pre-Treatment Prediction of Neoadjuvant Chemotherapy Response in Locally Advanced Gastric Cancer
Source: Diagnostics (Basel). 2026 Mar 23;16(6):945. doi: 10.3390/diagnostics16060945 (PMC13026075; doi:10.3390/diagnostics16060945)

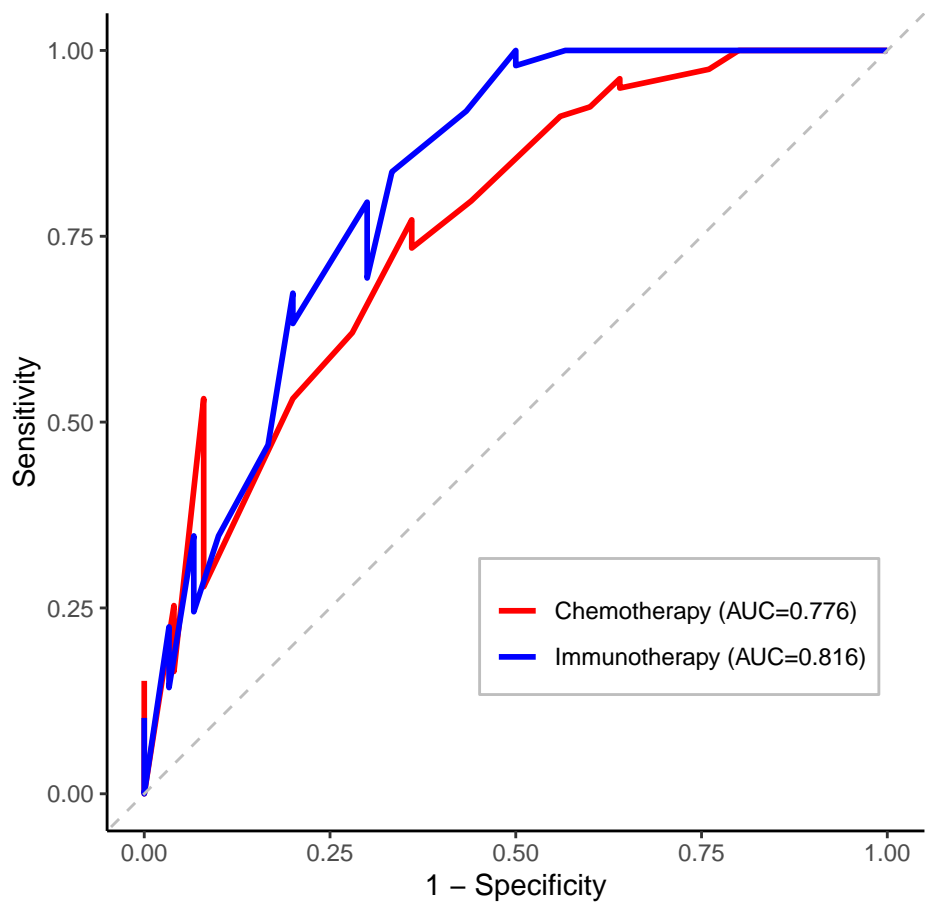

Supplement: Supplementary file 1 [file diagnostics-16-00945-s001.zip › Supplementary File/fig s1.pdf]
